# Interactions between Carotenoids from Marine Bacteria and Other Micronutrients: Impact on Stability and Antioxidant Activity

**DOI:** 10.3390/md13117020

**Published:** 2015-11-19

**Authors:** Charlotte Sy, Olivier Dangles, Patrick Borel, Catherine Caris-Veyrat

**Affiliations:** 1 INRA, UMR408 SQPOV, F-84000 Avignon, France; E-Mails: sy_charlotte@hotmail.com (C.S.); olivier.dangles@univ-avignon.fr (O.D.); 2 University of Avignon, UMR408 SQPOV, F-84000 Avignon, France; 3 INRA, UMR1260 NORT, F-13385 Marseille, France; E-Mail: patrick.borel@univ-amu.fr; 4 INSERM, UMR 1062, F-13385 Marseille, France; 5 Faculté de Médecine, Aix-Marseille Université, F-13385 Marseille, France

**Keywords:** carotenoids, marine bacteria, menaquinone, α-tocopherol, polyphenols, metmyoglobin, iron, oxidation, antioxidant activity

## Abstract

Recently isolated spore-forming pigmented marine bacteria *Bacillus indicus* HU36 are sources of oxygenated carotenoids with original structures (about fifteen distinct yellow and orange pigments with acylated d-glucosyl groups). In this study, we evaluated the stability (sensitivity to iron-induced autoxidation) and antioxidant activity (inhibition of iron-induced lipid peroxidation) of combinations of bacterial HU36 carotenoids with the bacterial vitamin menaquinone MQ-7 and with phenolic antioxidants (vitamin E, chlorogenic acid, rutin). Unexpectedly, MQ-7 strongly improves the ability of HU36 carotenoids to inhibit Fe^II^-induced lipid peroxidation, although MQ-7 was not consumed in the medium. We propose that their interaction modifies the carotenoid antioxidant mechanism(s), possibly by allowing carotenoids to scavenge the initiating radicals. For comparison, β-carotene and lycopene in combination were shown to exhibit a slightly higher stability toward iron-induced autoxidation, as well as an additive antioxidant activity as compared to the carotenoids, individually. HU36 carotenoids and phenolic antioxidants displayed synergistic activities in the inhibition of linoleic acid peroxidation induced by heme iron, but not by free iron. Synergism could arise from antioxidants interacting via electron transfer through the porphyrin nucleus of heme iron. Overall, combining antioxidants acting via complementary mechanisms could be the key for optimizing the activity of this bacterial carotenoid cocktail.

## 1. Introduction

Consumption of carotenoids may reduce the risk of developing chronic diseases associated with oxidative stress. Since carotenoids accumulate in much larger concentrations in the gastro-intestinal (GI) tract than in plasma and tissues, and because the GI tract may undergo substantial oxidative stress in postprandial conditions [1,2,3], part of this protection could take place prior to intestinal absorption [4,5,6]. For instance, dietary iron is highly present in food (especially in red meat), both as heme and in the free form [7,8]. Iron is a potent initiator of lipid peroxidation, especially in acidic conditions [9,10]. Indeed, under gastric conditions, dietary lipid peroxidation may be quite rapid due to high dioxygen concentrations, moderate temperature (37 °C), a pH varying between 2 (empty stomach) and 6 (postprandial), and constant mixing [5].

In solution and in organized lipid assemblies (e.g., micelles, liposomes, emulsions, and lipoproteins), carotenoids and other dietary antioxidants can interact. Previous studies have highlighted the possible regeneration of some antioxidants by others [11,12]. For instance, antioxidant synergism has been demonstrated using combinations of α-tocopherol, vitamin C, and β-carotene, as well as lutein [13,14,15,16,17,18,19,20]. The mechanisms proposed involve a transfer of electrons or hydrogen atoms between antioxidants partitioned between the aqueous and lipid phases via antioxidants located at the interface. A few studies have been conducted with combinations of carotenoids. For instance, in membranes, lycopene or β-carotene has been shown to significantly prolong the antioxidant activity of the xanthophyll zeaxanthin by reducing its radical, thereby restoring its active form [21].

In this work, we investigated possible mechanism(s) underlying the high stability and antioxidant activity of novel carotenoids extracted from a marine bacterial strain. *Bacillus indicus* HU36 was initially selected for its high production of carotenoids, the resistance of its spores to UV radiation [22], and its probiotic properties [23]. It was isolated from human feces and shown to synthesize yellow-orange pigments in variable proportions depending on whether the bacteria were present as vegetative cells or as spores [22]. The most abundant pigments in a lipophilic extract of vegetative cells were found to be 1-(6-C_n:0_)-glycosyl-apo-8′-lycopene esters, and in a lipophilic extract of spores were found to be methyl-1-(6-C_n:0_)-glycosyl-apo-8′-lycopenoate esters. Other isoprenoids were also observed [24]. In particular, menaquinone MQ-7, a form of vitamin K_2_, was extracted with the carotenoids in a crude extract. Menaquinones are constituents of bacterial cytoplasmic membranes and play important roles in electron transport, oxidative phosphorylation, active transport, and endospore formation [25]. As MQ-7 is potentially redox active, its influence on the stability and antioxidant activity of the bacterial carotenoids was tested. The common dietary carotenoids β-carotene and lycopene were also investigated for comparison.

Studies were carried out in micelle solutions [26], used as a simple model to mimic the environmental conditions experienced by dietary carotenoids in the GI tract [27,28]. Peroxidation was initiated either by free ferrous iron or by metmyoglobin, and followed at pH 5.8 and 4.0, corresponding to the early phase and mid-phase of digestion, respectively. The degradation of carotenoids and the formation of lipid oxidation products were followed by UV-VIS spectroscopy.

**Scheme 1:**
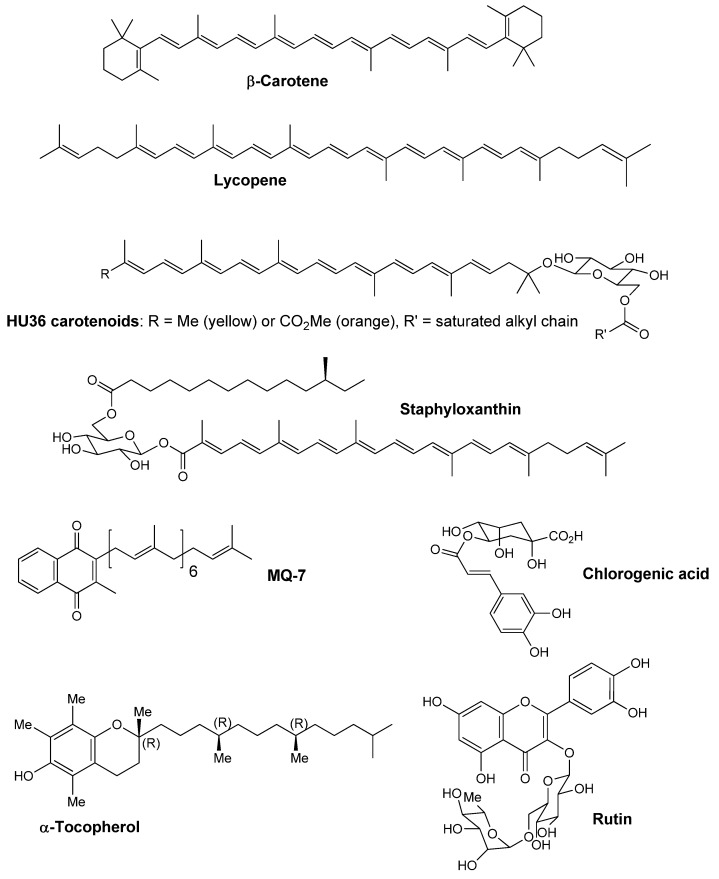
Chemical structures of studied molecules.

In a first step, the effect of the bacterial vitamin menaquinone MQ-7 (Scheme 1) on the stability and antioxidant properties of HU36 carotenoids was evaluated and compared with the effect on β-carotene. Then, combinations of carotenoids with similar structures (β-carotene and lycopene, Scheme 1) were evaluated to determine possible antioxidant interactions as compared to the cocktail of bacterial carotenoids. Finally, purified HU36 carotenoids were combined with other antioxidants (Scheme 1) in order to suggest formulations that could enhance their stability and antioxidant properties.

## 2. Results

### 2.1. Composition of the HU36 Carotenoid Extract

Ultra-high performance liquid chromatography-mass spectrometry (UPLC-MS) analyses of the crude bacterial extract containing HU36 carotenoids were in agreement with the carotenoid composition described in the literature [24]. Two carotenoid glucosides were identified as the parent structures of all subsequent peaks: *O*-glycosyl carotenoid and methylester *O*-glycosyl carotenoid (Scheme 1). The free form of each carotenoid eluted first, followed by their corresponding fatty acid esters, with each additional peak differing only in terms of the secondary fatty acid chain to which the parent structures were acylated. Five compounds were identified corresponding to *O*-glycosyl carotenoid, a yellow pigment with a fine three-prong UV-VIS structure absorbing at 429, 454 (λ_max_), and 484 nm. Likewise, a set of eight compounds were identified with a parent structure of methylester *O*-glycosyl carotenoid, an orange pigment with a fine three-prong UV-VIS structure absorbing at 445, 468 (λ_max_), and 496 nm. The analyses also revealed more hydrophobic isoprenoids, e.g., the last major peak was attributed to MQ-7, a menaquinone with seven isoprenyl subunits (Supplementary Figure S1).

After purification of ~250 mg of crude extract, 3.5 μmol of pure HU36 carotenoids (mixture of yellow and orange pigments) and 20 μmol of MQ-7 were obtained.

### 2.2. Influence of MQ-7 on the Stability and Antioxidant Properties of Carotenoids

*Stability and antioxidant activity of MQ-7*. MQ-7 is more efficient globally against lipid peroxidation at pH 5.8 than at pH 4. However, it has much lower antioxidant activity as compared to carotenoids, with very high IC_50_ values: 30.5 ± 1.0 μmol·L^−1^ with MbFe^III^ at pH 5.8, 59.4 ± 8.5 μmol·L^−1^ with MbFe^III^ at pH 4, and 47.4 ± 5. 4 μmol·L^−1^ with Fe^II^ at pH 4. From the shape of kinetic curves (data not shown), MQ-7 slightly prolongs the oxidation induction period and slows down propagation. Contrary to carotenoids, MQ-7 is not degraded in the presence of Fe^II^ (data not shown).

*Stability of HU36 bacterial carotenoid extracts toward iron.* The stability of the crude HU36 extract (e.g., in the presence of MQ-7) was compared with that of the purified HU36 extract (e.g., in the absence of MQ-7).

After 4 h at pH 4 in the absence of iron, carotenoid concentrations remained almost constant in both samples. The slight degradation observed was associated with “spontaneous” autoxidation (initiated by unidentified metal traces present in the mixture). After 4 h of reaction with iron, the most intense degradation was observed with an iron/carotenoid molar ratio of *ca.* 5 for Fe^II^, 10 for Fe^III^, and 0.05 for heme iron (MbFe^III^). Both samples were very stable toward MbFe^III^, with only 10% consumption after 4 h. Carotenoid degradation after 4 h was more pronounced when oxidation was initiated by Fe^III^ (*ca.* 50% degradation) as compared to Fe^II^ (*ca.* 30% degradation) (Supplementary Figure S2). No significant difference in stability was observed between crude and purified bacterial carotenoids in the presence of Fe^II^ or MbFe^III^. In the presence of Fe^III^, purified carotenoids were found to be slightly less stable than the crude extract containing MQ-7.

**Figure 1:**
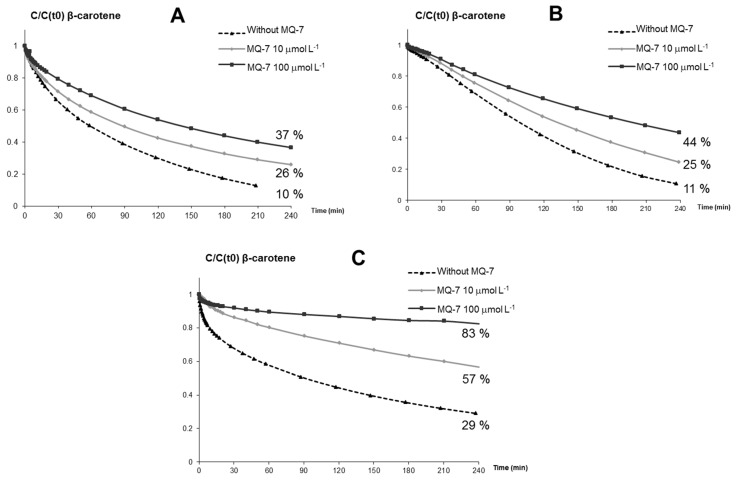
Iron-induced autoxidation of β-carotene (10 μmol·L^−1^) without and with MQ-7 (10 and 100 μmol·L^−1^). Kinetic monitoring at pH 4 after addition of (**A**) 50 μmol·L^−1^ Fe^II^; and (**B**) 100 μmol·L^−1^ Fe^III^ and (**C**) 0.5 μmol·L^−1^ MbFe^III^ as oxidation initiators. Percentages represent the final concentration of β-carotene remaining at the end of the experiment relative to *t* = 0 min.

*Influence of MQ-7 on the stability of β-carotene in the presence of iron.* The stability of β-carotene was compared in the presence and absence of purified MQ-7 at two concentrations (10 and 100 μmol·L^−1^). The kinetic profiles of the curves were similar, but MQ-7 lowered β-carotene degradation, especially in the presence of heme iron (Figure 1A–C). Stability of β-carotene toward heme-induced autoxidation was increased two-fold with 1 equiv. MQ-7 and three- to four-fold with 10 equiv. MQ-7.

**Figure 2:**
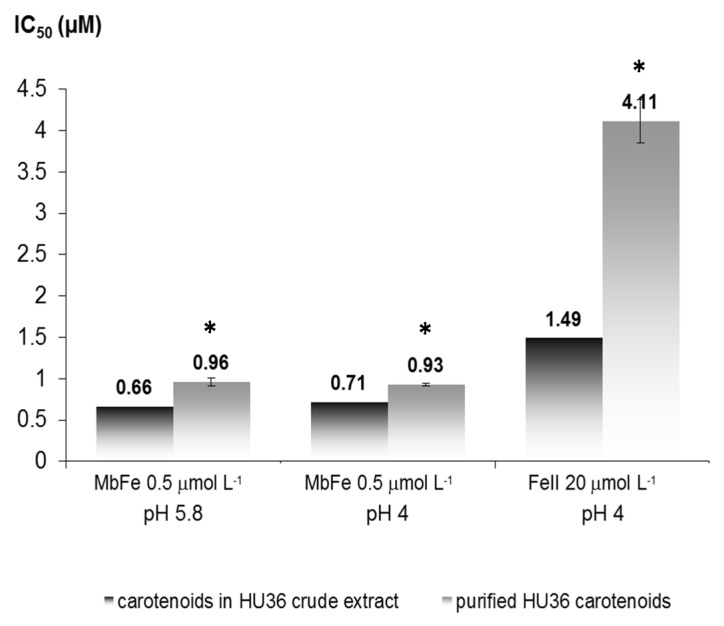
IC_50_ values for the inhibition of iron-induced linoleic acid peroxidation by crude and purified HU36 carotenoids (e.g., with or without MQ-7, respectively). Oxidation was initiated with 0.5 μmol·L^−1^ MbFe^III^ or 20 μmol·L^−1^ Fe^II^ (as noted in the figure). * indicates a significant difference between the crude and purified IC_50_ values within the same treatment (student *t* test, *p* < 0.05). For the evaluation of IC_50_ values, see the Experimental Section.

*Antioxidant activity of crude and purified HU36 bacterial carotenoid extracts.* From the T/T_0_ *vs.* antioxidant concentration plots, IC_50_ values (T/T_0_ = 0.5) were extracted and used to quantify the antioxidant capacity [12,26] (Figure 2); the lower the IC_50_ value, the more efficient the antioxidant. In the three models of linoleic acid peroxidation, the purified carotenoid extract was found to provide significantly less antioxidant protection than the extract containing MQ-7 (Figure 2). More specifically, the antioxidant capacity of the crude extract was increased by 45% in MbFe^III^-induced peroxidation at pH 5.8 and by 30% at pH 4. Remarkably, the antioxidant capacity of the crude extract was more than twice as high in Fe^II^-induced peroxidation at pH 4 as compared to the purified extract.

**Figure 3:**
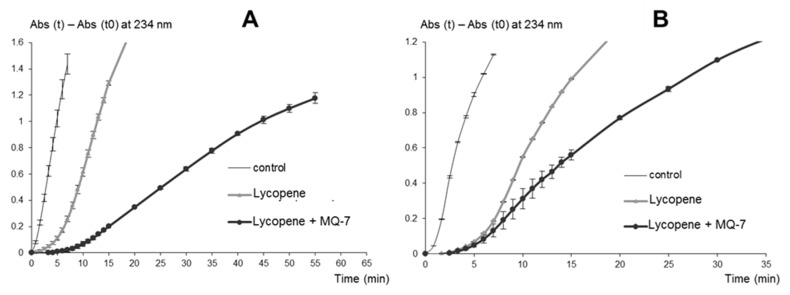

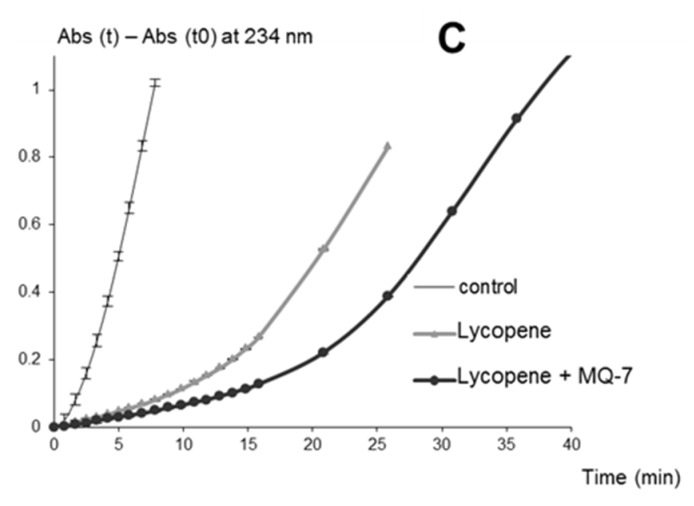
Kinetic monitoring of conjugated diene (CD) accumulation during linoleic acid peroxidation inhibited by lycopene (2 μmol·L^−1^) and lycopene + menaquinone (MQ-7, 20 μmol·L^−1^). Initiation by (**A**) 0.5 μmol·L^−1^ MbFe^III^, pH 5.8; (**B**) 0.5 μmol·L^−1^ MbFe^III^, pH 4 and (**C**) 20 μmol·L^−1^ Fe^II^, pH 4.

*Influence of MQ-7 on the antioxidant activity of lycopene.* Lycopene is a potent inhibitor of linoleic acid peroxidation initiated by Fe^II^ or MbFe^III^ [29]. The delays of linoleic acid peroxidation induced by 2 μmol·L^−1^ lycopene with and without MQ-7 (10 equiv.) were compared. The molar ratio of MQ-7/lycopene was in the same range as that of MQ-7/carotenoid in the crude HU36 extract. When lycopene and MQ-7 were combined, the delay in linoleic acid peroxidation was three times (Figure 3A), 1.6 times (Figure 3B), and 1.3 times (Figure 3C) higher than lycopene alone, with an initiator of MbFe^III^ at pH 5.8, MbFe^III^ at pH 4, and Fe^II^ at pH 4, respectively. Thus, the effect of MQ-7 on lycopene activity was maximal with MbFe^III^ at pH 5.8 and minimal with Fe^II^ at pH 4, in sharp contrast with the HU36 carotenoids.

### 2.3. Stability and Antioxidant Activity of Combinations of Carotenoids

In comparison with lycopene and β-carotene, bacterial carotenoids were found to be significantly more stable in all models [30], and better inhibitors of MbFe^III^-induced lipid peroxidation [31]. However, it was not clear whether these properties reflected the intrinsic stability and antioxidant activity of the individual carotenoids, or whether interactions between components of the mixture were important. In complementary experiments, lycopene and β-carotene were tested simultaneously in order to evaluate their possible interactions.

**Figure 4:**
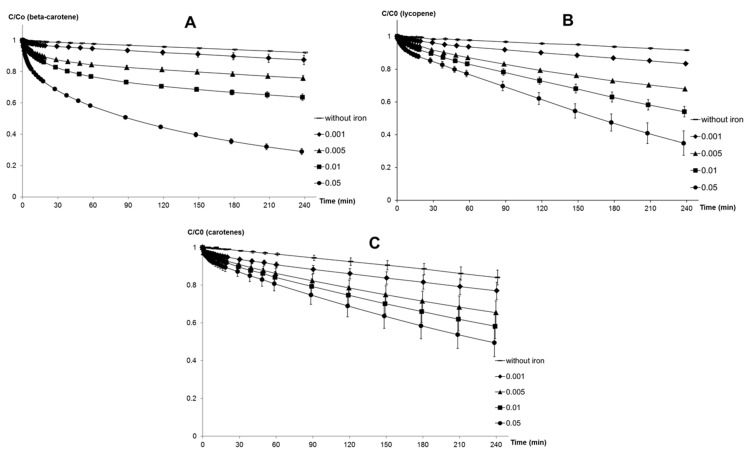
Percentage of residual β-carotene (**A**); lycopene (**B**); and lycopene + β-carotene (carotenes) (**C**) during MbFe^III^-induced autoxidation at pH 4. MbFe^III^/carotene molar ratio = 0, 0.001, 0.005, 0.01, and 0.05.

*Stability of β-carotene and lycopene, alone and in combination.* In the micelle model at pH 4, oxidation of β-carotene and lycopene alone (10 μmol·L^−1^ each) was compared with the oxidation of the mixture in equimolar concentrations (total concentration: 10 μmol·L^−1^, λ_max_ = 480 nm). Whatever the form and iron concentration, β-carotene and lycopene were more stable in combination with one another (Figure 4 and Supplementary Figures S3 and S4).

*Antioxidant activity of β-carotene and lycopene alone and in combination.* Linoleic acid peroxidation was inhibited by β-carotene, lycopene, and by a 1:1 mixture of both carotenes at the same total concentration (Supplementary Figure S5). Regardless of the iron initiator (MbFe^III^ or Fe^II^) and the pH (5.8 or 4), the induction period of CD accumulation was longer with lycopene than with β-carotene, confirming that lycopene is a better antioxidant than β-carotene in this assay. Moreover, in all experiments, the equimolar mixture of lycopene and β-carotene displayed an intermediate behavior corresponding to an average of single antioxidant activities. Thus, the antioxidant activities of lycopene and β-carotene are additive and no synergism is observed.

### 2.4. Interactions between Carotenoids and Phenolic Antioxidants

**Figure 5:**
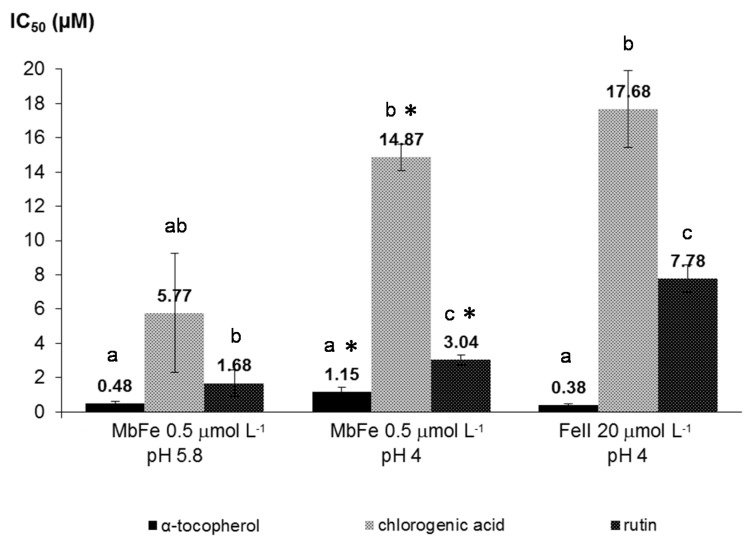
IC_50_ values for the inhibition of iron-induced linoleic acid peroxidation by α-tocopherol, chlorogenic acid, and rutin. Different letters indicate a significant difference between the IC_50_ values within the same experimental condition (ANOVA followed by the Tukey-Kramer *post hoc* test, *p* < 0.05). * indicates a significant difference between the IC_50_ value of the same antioxidant compared at pH 4 and 5.8 with MbFe as the initiator (Student’s *t* test, *p* < 0.05).

*Antioxidant capacities of phenolic antioxidants.* The ability of α-tocopherol and two common dietary phenols, rutin and chlorogenic acid, to inhibit lipid peroxidation was first studied with individual antioxidants. Considering the curves of CD accumulation (data not shown), it is clear that α-tocopherol resulted in a longer induction period, which is consistent with the well-known activity of α-tocopherol as a chain-breaking antioxidant by reducing the propagating LOO^•^ radicals [26]. In contrast, while rutin and chlorogenic acid did not prolong the induction period, they did continuously slow peroxidation. This phenomena is typical of antioxidants located in the aqueous phase and/or at the interface which act by reducing hypervalent heme iron or initiating radicals [12]. In all cases, the antioxidant hierarchy, based on the IC_50_ values, was: α-tocopherol > rutin ≥ chlorogenic acid (Figure 5). Moreover, all three antioxidants were more efficient against lipid peroxidation initiated by MbFe^III^ at pH 5.8 than at pH 4.

*Impact of phenolic antioxidants on the antioxidant activity of HU36 carotenoids.* Each antioxidant was used at a concentration close to its IC_50_ value: 1 μmol·L^−1^ for the purified HU36 carotenoid extract, 0.4 μmol·L^−1^ for α-tocopherol, 15 μmol·L^−1^ for chlorogenic acid, and 5 μmol·L^−1^ for rutin. In each model, CD accumulation was determined in the presence of HU36 carotenoids alone, the selected phenol alone, and a combination of both HU36 carotenoids and the phenol. Then, a simulated curve assuming additive behavior was constructed as follows: time period for accumulation of any given CD concentration = T_0_ (no antioxidant), T_1_ = T_0_ + ΔT_1_ (antioxidant 1), T_2_ = T_0_ + ΔT_2_ (antioxidant 2), T_1+2_^add^ = T_0_ + ΔT_1_ + ΔT_2_ (antioxidant 1 + antioxidant 2, assuming additivity).

Finally, interactions between antioxidants were evaluated by comparing the simulated and experimental peroxidation curves of antioxidant mixtures: additivity if the curves approximately coincide (T_1+2_^exp^ ≈ T_1+2_^add^), synergy if the experimental curve was shifted toward longer delays (T_1+2_^exp^ > T_1+2_^add^), and antagonism in the opposite case (T_1+2_^exp^ < T_1+2_^add^). For a fixed CD concentration (corresponding to ΔA(234 nm) = 0.5), a percentage of synergy or antagonism can be evaluated (Table 1):

% synergy or antagonism = 100 × (T_1+2_^exp^ − T_1+2_^add^)/T_1+2_^add^

**Table 1:** Interactions between HU36 carotenoids (1 μmol·L^−1^) and phenolic antioxidants during iron-initiated (0.5 μmol·L^−1^ MbFe^III^, 20 μmol·L^−1^ Fe^II^) linoleic acid peroxidation.

	% of Synergy (+) or Antagonism (−) *		
	MbFe^III^ pH 5.8	MbFe^III^ pH 4	Fe^II^ pH 4
HU36 + α-tocopherol (0.4 equiv.)	-	-	−44.4
HU36 + chlorogenic acid (15 equiv.)	+118.5	+101.6	−42.8
HU36 + rutin (5 equiv.)	+105.3	+39.0	−42.3

* See text for calculations.

**Figure 6:**
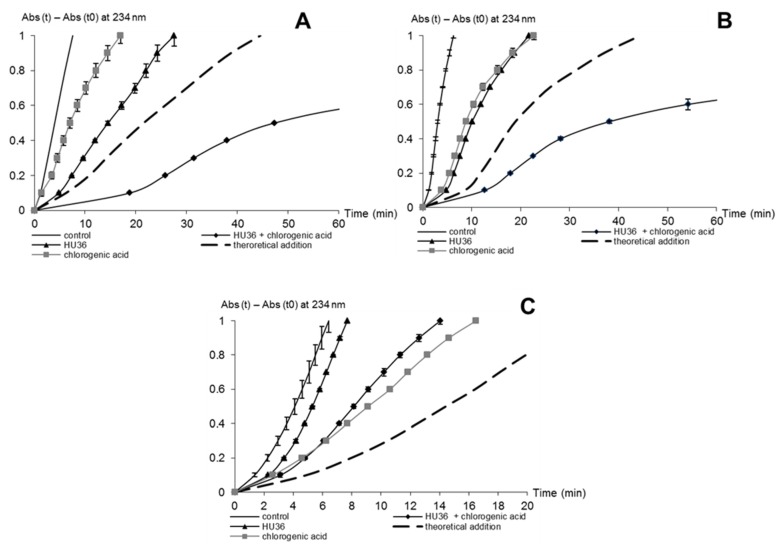
CD accumulation during linoleic acid peroxidation inhibited by 1 μmol·L^−1^ HU36 carotenoids, by 15 μmol·L^−1^ chlorogenic acid, and by a combination of the two antioxidants at the same concentration. Initiation by MbFe^III^, pH 5.8 (**A**) MbFe^III^, pH 4 (**B**) and Fe^II^, pH 4 (**C**). Dashed curve: simulation assuming additive antioxidant behaviors.

In the inhibition of MbFe^III^-initiated peroxidation, synergistic interactions were highlighted between HU36 carotenoids and the two phenols, but not between HU36 carotenoids and α-tocopherol (where additivity was observed) (Figure 6). In contrast, antagonistic effects were observed when lipid peroxidation was induced by Fe^II^.

### 2.5. Reduction of Ferrylmyoglobin by Antioxidants

When MbFe^III^ is treated with H_2_O_2_ (0.5 equiv.) in the absence of antioxidant, formation of ferrylmyoglobin (MbFe^IV^=O) can be followed by monitoring 590 nm (Supplementary Figure S6) [26]. The reaction was carried out at pH 7 to improve MbFe^IV^=O stability to facilitate its investigation. MbFe^IV^=O is a potent initiator of lipid peroxidation [9,32], although its role in the absence of added H_2_O_2_ may be questioned [33]. A decay of the absorbance at 590 nm following the addition of an antioxidant is observed if the antioxidant is able to enter the heme cavity and reduce the iron-oxo group to regenerate MbFe^III^. In the presence of α-tocopherol, reduction of ferrylmyoglobin was slow and indistinguishable from that observed with MeOH alone (e.g., in the control experiment). In the presence of β-carotene or lycopene, the carotenoids slightly stabilized MbFe^IV^=O as compared to control. The addition of HU36 carotenoids induced a slight reduction of MbFe^IV^=O to MbFe^III^. These results demonstrate that the lipophilic antioxidants tested in this model, carotenoids and α-tocopherol, do not significantly react with hypervalent iron. By contrast, chlorogenic acid and rutin [34] markedly reduce MbFe^IV^=O, suggesting that these hydrophilic antioxidants can reach the heme in the aqueous medium and quickly reduce the peroxidation initiator. The observation of distinct behaviors for lipophilic and hydrophilic antioxidants in the inhibition of the metmyoglobin-induced peroxidation of linoleic acid is in agreement with the literature [12,34].

## 3. Discussion

The stability and lipid-protecting capacity of antioxidant mixtures were investigated in this work using the following combinations: mixtures of carotenoids, carotenoids + MQ-7, and carotenoids + phenolic antioxidants.

MQ-7 does not significantly inhibit iron-induced autoxidation of HU36 carotenoids (a weak protection was observed only with Fe^III^). However, the protective effect of MQ-7 is very significant with β-carotene, especially in the presence of heme iron. Thus, protection is very dependent on the form of iron and the type of carotenoid. Although a poor antioxidant compared to HU36 carotenoids and lycopene, MQ-7 in relatively high concentrations (10 molar equiv.) can efficiently increase the antioxidant activity of carotenoids. This effect is maximally observed in Fe^II^-induced lipid peroxidation with the bacterial carotenoids, and in heme-induced peroxidation with lycopene. However, MQ-7 is not degraded in the presence of iron.

**Scheme 2:**
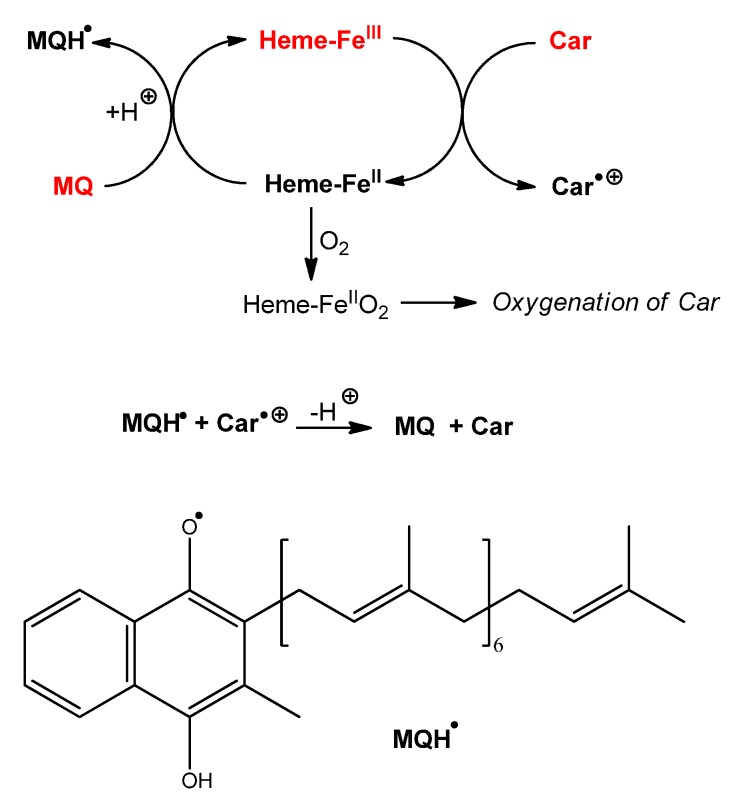
Proposed mechanisms of protection of carotenoids by menaquinone during heme-induced lipid peroxidation.

Although not an electron-donor by itself, MQ-7 (abbreviated as MQ below and in Scheme 2) may be involved in redox cycling through its one-electron reduced (semiquinone, MQH^•^) and two-electron reduced (hydroquinone, MQH_2_) forms [35,36]. Such species could be generated by electron transfer from iron—carotenoid systems to MQ depending on the location and reducing capacity of carotenoids. MQH^•^ and MQH_2_ could in turn effectively transfer electrons to lipid-derived radicals, carotenoid radical cations, and/or hypervalent heme-iron species involved in the initiation of lipid peroxidation.

For instance, from the mechanism of heme-induced autoxidation of carotenoids previously proposed [30], a mechanism for the protection of carotenoids by MQ-7 can be suggested (Scheme 2). By sparing carotenoids, MQ-7 is also susceptible to increase their apparent antioxidant efficiency in inhibiting heme-induced lipid peroxidation. However, this mechanism is likely limited to carotenes (β-carotene, lycopene).

**Scheme 3:**
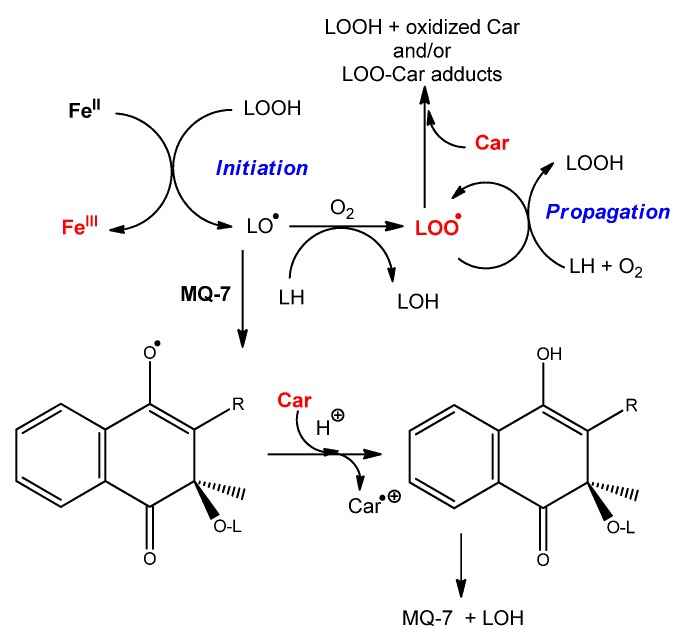
Proposed mechanism for the inhibition of Fe^II^-induced lipid peroxidation (from [31]) by carotenoids, and interaction of marine bacterial carotenoids with menaquinone MQ-7.

The observation that MQ-7 strongly improves the ability of HU36 carotenoids to inhibit Fe^II^-induced lipid peroxidation is unexpected. MQ-7 does not react with iron and is a poor antioxidant by itself, which makes it unlikely that it could directly quench propagating lipid peroxyl radicals (LOO^•^). The previously proposed mechanism for the inhibition of Fe^II^-induced lipid peroxidation by carotenoids is reintroduced in Scheme 3 [31]. A possible interpretation of the effect of MQ-7 is that the initiating oxyl radical(s) (*i.e.*, LO^•^, or other radicals derived from LO^•^) could combine with MQ-7 to form a stabilized radical, which could then be rapidly reduced by the carotenoids. In other words, HU36 carotenoids would be able to interfere in the initiation process via MQ-7, in addition to their conventional action on the propagation step (LOO^•^ scavenging).

When used in combination, β-carotene and lycopene exhibit a slightly greater stability toward iron-induced autoxidation than the individual carotenoids in equivalent concentrations. It is possible that each carotenoid may be protected by the radical-scavenging activity of the other. However, HPLC analysis would be required to reveal whether one carotenoid is more significantly spared than the other. Stability of the cocktail of HU36 carotenoids might be reinforced by similar interactions.

A mixture of lycopene and β-carotene displays the same efficiency at inhibiting lipid peroxidation as that expected from the activity of the individual pigments (additivity). Thus, the antioxidant activity of HU36 carotenoids might also correspond to the additive contribution of the individual carotenoids in the cocktail.

Combinations of HU36 carotenoids and phenolic antioxidants displayed synergistic activities in the inhibition of linoleic acid peroxidation induced by heme iron, but not by free iron. Phenolic antioxidants may act in synergy with HU36 carotenoids, either by protecting them from oxidation during lipid peroxidation, or by acting through a complementary antioxidant mechanism. Simultaneous monitoring of carotenoid consumption showed that α-tocopherol and the polyphenols tested did not significantly protect the carotenoids in the presence of iron (data not shown). Thus, synergism cannot be explained by a recycling of the bacterial carotenoids by the phenolic antioxidants.

**Scheme 4:**
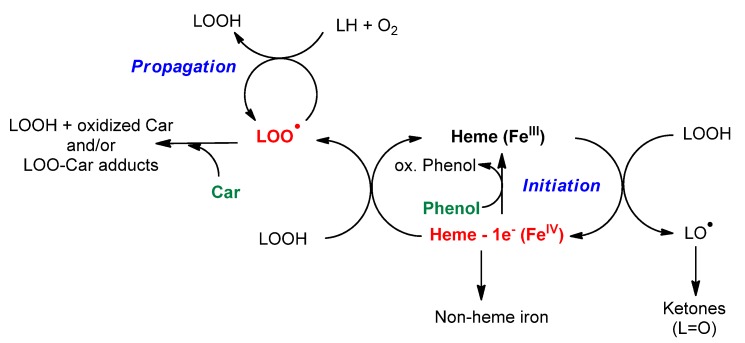
Proposed mechanism for the complementary antioxidant activity of marine bacterial carotenoids and phenolic compounds during heme-induced lipid peroxidation.

Complementary experiments have highlighted that chlorogenic acid and rutin rapidly reduce ferrylmyoglobin (Fe^IV^), a potential initiator of lipid peroxidation, while HU36 carotenoids and α-tocopherol are essentially inactive. On the other hand, α-tocopherol and the bacterial carotenoids can act as chain-breaking antioxidants by direct scavenging of the propagating lipid peroxyl radicals in the lipid phase [26]. Thus, complementary antioxidant mechanisms that may result in synergy can be suggested for the polyphenols and bacterial carotenoids (Scheme 4). The heme cofactor of metmyoglobin was already proposed as a key component of synergism between α-tocopherol and quercetin [12]. In this previously reported study, it was demonstrated that α-tocopherol, while unable to reduce the iron-oxo center of ferrylmyoglobin, can protect the porphyrin nucleus from oxidative degradation (as revealed by the decay of the Soret band). Moreover, quercetin, which can quickly reduce MbFe^IV^=O, is partially spared when quercetin + α-tocopherol mixtures are used for the inhibition of lipid peroxidation (while α-tocopherol consumption is enhanced). Hence, the synergism observed was attributed to a regeneration of quercetin (and/or some of its oxidation products with a residual antioxidant activity) from its radical by α-tocopherol via electron transfer through the porphyrin nucleus. A similar mechanism may apply between polyphenols and the bacterial carotenoids.

## 4. Experimental Section

### 4.1. Chemicals

Natural (all-*E*) lycopene from tomato oleoresin (C_40_H_56_, M = 536 g·mol^−1^, >90%) was obtained from Conesa, Badajoz, Spain. Carotenoid extracts from *Bacillus* strains HU36 were provided by members of the Colorspore consortium (Small Collaborative Project No. 207948, funded by FP7 and coordinated by the Royal Holloway University of London) [37]. Synthetic type II (all-*E*) β-carotene (>95%), α-tocopherol (95%), rutin (≥95%), chlorogenic acid (≥95%), menaquinone K2 (MK-4, ≥99.9%), polyoxyethyleneglycol 23 lauryl ether (Brij^®^35), (9*Z*,12*Z*)-octadecadienoic acid (linoleic acid, >99%), FeSO_4_·7H_2_O (>99.5%), Fe(NO_3_)_3_·9H_2_O (98%), myoglobin from equine heart (>90%, essentially salt-free), and ammonium formate (99.995%) were purchased from Sigma-Aldrich (St-Quentin-Fallavier, France). The buffers used in the experiments were a 0.02 mol·L^−1^ acetate buffer (pH 4.0) and a 0.2 mol·L^−1^ phosphate buffer (pH 5.8). Water was purified through a Millipore Q-Plus apparatus.

Acetic acid (100%), formic acid (>98%) and LC-MS grade methanol (MeOH) were purchased from Merck (Darmstadt, Germany). All other solvents were from Fisher-Scientific (Loughborough, UK). Dichloromethane (CH_2_Cl_2_) and acetonitrile (MeCN) were of UPLC grade, and ethylacetate (EtOAc) was of analytical grade.

### 4.2. Purification of the Bacterial Carotenoid Extracts

*Fractionation of crude bacterial extracts by flash liquid chromatography*. The crude bacterial extracts from HU36 were dissolved in CH_2_Cl_2_/MeOH (1/1, v:v), filtered through glass cotton to eliminate residues of the culture medium, and dried by evaporation under reduced pressure. For purification, about 250 mg of dry crude extract was dissolved in the smallest volume of MeOH and placed in the top a burette, (30 cm length, 2 cm diameter) filled with C-18 silica gel column 47 μm × 60 μm A.P.S. (DAVISIL^®^ Chromatographic Silica Media for separation and purification applications, grade 633N, W.R. Grace Columbia, MD, USA) and containing a sintered glass filter. The C-18 silica gel was previously moistened with MeOH, 1% aq. CH_3_CO_2_H, and the column was rinsed with MeOH/H_2_O (80:20, v/v). The elution of the content of the bacterial extract was performed with a two solvent-gradient and the flow rate was maintained regular with a flow of argon. Solvent A was MeOH/H_2_O (80:20, v/v) and solvent B was EtOAc/CH_2_Cl_2_ (80:20, v/v). The gradient was: 250 mL A 100%, 75 mL A 90% B 10%, 75 mL A 85% B 15%, 75 mL A 80% B 20%, 100 mL A 75% B 25%, 250 mL A 65% B 35%, 75 mL A 50% B 50%, 50 mL de A 40% B 60%, 75 mL A 20% B 80%, 50 mL B 100%. The elution fractions were progressively collected in 25 mL glass tubes. A sample of each fraction was filtered through a 20 μm PTF filter and analyzed by UPLC-MS to ensure the absence of menaquinone in colored fractions and the presence of menaquinone in the last uncolored fraction. After each new purification, the C-18 silica gel was regenerated by several washings with Milli-Q water and MeOH/CH_2_Cl_2_ (1:1, v/v).

*Structural analysis of the fractions.* Reversed-phase ultra-fast liquid chromatography analyses were performed on a Waters Acquity™ Ultra Performance LC^®^ system equipped with a diode array detector and coupled to an esquire HCT™ (high capacity trap) ultra-MS^®^ mass spectrophotometer (Bruker-Daltonics). A Waters Acquity™ reversed-phase C_18_ column type HSS T3 (2.1 mm× 150 mm, 1.8 μm particle size) was used to separate fraction components. For the separation, gradient elution was performed at 25 °C, using a two solvent mobile phase at a flow rate of 0.5 mL·min^−1^. Eluent A contained 5 μmol·L^−1^ HCO_2_H and 10 mmol·L^−1^ HCO_2_NH_4_ in a mixture of MeCN/MeOH/H_2_O (60:20:20, v/v/v). Eluent B contained 5 μmol·L^−1^ HCO_2_H in EtOAc/CH_2_Cl_2_ (80:20, v/v). The gradient was as follows: 0% B held for 2 min, linearly increasing to 55% B over 14.5 min, holding at 55% B for 0.5 min, increasing to 60% B over 7 min, increasing to 70% B over 3 min, holding at 70% B for 1 min, returning to 0% B over 0.01 min, and holding at 0% B for 2 additional minutes (to rinse the system and return the column to the initial conditions before the following injection). Absorbance was followed by UV-VIS detection every 2 nm from 250 to 800 nm, with a time interval of 0.1 min. The effluent of the column was interfaced with the ion source of the MS using APCI (atmospheric pressure chemical ionization) alternating between positive and negative mode. The additional MS parameters were as follows: corona intensity = 1 μA, cone pressure = 40 psi, dry gas flow rate = 4 L·min^−1^ at a temperature of 300 °C. Spectra were acquired every 40 ms with a mass range of 50 to 1000 *m*/*z*. For optimal detection, windows were drawn to focus on target masses throughout the analysis. A *m*/*z* of 350 was targeted for *t* = 0–7 min, *m*/*z* 600 for *t* = 7–11.5 min, and *m*/*z* 750 for *t* = 11.5–26 min. In each fraction, *m*/*z* = 649.3 corresponding to MQ-7 was searched. Fractions were collected, gathered, and the total volume was measured and evaporated under reduced pressure. Finally, a pure bacterial carotenoid stock solution was obtained by dissolving the dry residue in MeOH/CH_2_Cl_2_ (1:1, v/v).

*Quantification of purified bacterial carotenoids and the menaquinone content of the initial crude extract.* Before evaporation, a sub-sample of the total volume of collected fractions was evaporated under reduced pressure, and dissolved it in the same volume of CH_2_Cl_2_ for spectrophotometric measurement. Absorbance was recorded at the respective λ_max_ for the particular parent carotenoid (previously described in the introduction) and the fraction concentration was calculated using the previously determined molar absorption coefficient (ε = 165 × 10^3^ M^−1^·cm^−1^ at 454 nm) [31]. Total extracted MQ-7 was quantified by UPLC-MS using an external calibration curve established with commercially available menaquinone.

### 4.3. Chemical Models of Antioxidant Activity in the Gastric Compartment

*Stock solutions of antioxidants.* Four stock carotenoid solutions (~1 mmol·L^−1^) were used: a crude bacterial extract from HU36, purified bacterial carotenoids extract from HU36, (all-*E*) lycopene, and (all-*E*) β-carotene. Bacterial extracts were dissolved in CH_2_Cl_2_/MeOH (1:1, v/v) and lycopene and β-carotene in CH_2_Cl_2_. The concentrations of stock carotenoid solutions were calculated by spectrophotometric measurement. The molar absorption coefficients used were 128.5 × 10^3^ L·mol^−1^·cm^−1^ at 460 nm for β-carotene, 178 × 10^3^ L·mol^−1^·cm^−1^ at 482 nm for lycopene and 165 × 10^3^ L·mol^−1^·cm^−1^ at 454 nm for HU36 carotenoids in CH_2_Cl_2_ [38].

A 232 μmol·L^−1^ α-tocopherol solution, a 1 mmol L^−1^ chlorogenic acid solution, and a 350 μmol·L^−1^ rutin solution were also prepared by dissolving 1.00 mg, 3.54 mg, and 2.74 mg, respectively, in 10 mL MeOH.

*Micelle solution.* Both stability and antioxidant tests were performed using a model adapted from a previous study [26]. Experiments were performed at 37 °C, pH 4 (0.2 mol·L^−1^ acetate buffer) and pH 5.8 (0.2 mol·L^−1^ phosphate buffer), mimicking the two stages of digestion in humans [27]. The synthetic non-ionic hydroperoxide-free surfactant Brij^®^35 (polyoxyethyleneglycol dodecyl ether) was selected for the preparation of carotenoid micelles in the aqueous medium [39]. Brij^®^35 was dissolved in CH_2_Cl_2_ to obtain a 40 mmol·L^−1^ stock solution. Linoleic acid was added only for antioxidant tests (not for stability tests). A 28 mmol·L^−1^ stock solution was prepared by dissolving 39.25 mg commercial linoleic acid in 5 mL CH_2_Cl_2_.

Aliquots of stock carotenoid solutions and/or other antioxidant solutions were dispersed with 2 mL stock surfactant solution and 0 or 250 μL linoleic acid stock solution. After evaporation of the solvent under reduced pressure and dilution of the dried viscous residue in 20 mL aqueous buffer, initial concentrations were 4 mmol·L^−1^ Brij^®^35 and 10 μmol·L^−1^ carotenoid for stability tests; 4 mmol·L^−1^ Brij^®^35, 0.7 mmol·L^−1^ linoleic acid, 0 to 8 μmol·L^−1^ carotenoid, and/or 0 to 100 μmol·L^−1^ of another antioxidant for the antioxidant activity experiments. Micelles were formed by stirring the solution on a magnetic stir plate until total homogenization. The micelle solution was perfectly transparent.

*Iron solutions*. Oxidation was initiated by Fe^II^, Fe^III^, or metmyoglobin (MbFe^III^, heme iron).

Stock 0.5 mol·L^−1^ Fe^III^ solution was prepared by dissolving 404 mg ferric nitrate nonahydrate in 2 mL 0.1 mol·L^−1^ nitric acid, stock 0.5 mol·L^−1^ Fe^II^ solution was prepared by dissolving 279.4 mg ferrous sulphate heptahydrate in 2 mL 0.1 mol·L^−1^ sulphuric acid and stock 0.5 mmol·L^−1^ MbFe^III^ solution was prepared by dissolving 8.9 mg myoglobin from equine heart in 1 mL Milli-Q water. Due to the poor solubility of myoglobin, the stock solution was filtered and its concentration was checked by absorbance measurement at 505 nm, with ε = 97 × 10^3^ L·mol^−1^·cm^−1^ [40]. Dilute iron solutions were then prepared from the stock solution. For stability tests, seven Fe^II^ solutions (0.5–25 mmol·L^−1^) were prepared in sulfuric acid, seven Fe^III^ solutions (0.05–25 mmol·L^−1^) were prepared in nitric acid, and seven MbFe solutions (0.5–500 μmol·L^−1^) were prepared in milliQ water. For antioxidant tests, only 1 mmol·L^−1^ Fe^II^ and 25 μmol·L^−1^ MbFe^III^ were used.

### 4.4. Stability Study and Lipid Peroxidation Inhibition

*UV-VIS Spectroscopy*. All experiments were performed in glassware protected from light, covered with Teflon stoppers, and kept under magnetic stirring at a constant temperature of 37 °C. Spectra were recorded on a Specord S-600 diode-array spectrophotometer (optical path length = 1 cm), equipped with an eight-cell rail, a magnetic stirring device, and a thermostatic bath (Analytik Jena AG, Jena, Germany). Each treatment was run in triplicate.

*Kinetics of carotenoid oxidation in micelles*. Stability measurements were performed at pH 4 and 37 °C. 1.96 mL of the micelle solution containing 4 mmol·L^−1^ Brij^®^35 and 10 μmol·L^−1^ carotenoids alone or in combination were transferred to a macro quartz spectrophotometer cell. At time zero, oxidation was initiated by addition of 40 μL iron solution to the reaction medium. Oxidation was initiated by iron concentrations in the range of 1–1000 μmol·L^−1^ Fe^II^ and Fe^III^ or 0.01–10 μmol·L^−1^ MbFe^III^. Residual carotenoid concentrations were measured directly by UV-VIS spectrometry (normalized to a baseline of acetate buffer alone).

Spectra were recorded at regular intervals for 4 h, from 300 to 1000 nm, and kinetic curves were plotted by extracting the absorbance at the wavelength maximum of each respective carotenoid sample. Results were expressed as a % relative to the initial carotenoid content:

% residual carotenoid = [Abs(λ_max_)_t_/Abs(λ_max_)_t0_] × 100

*Antioxidant activity in the micelle solution.* 1.96 mL of the micelle solution, containing 4 mmol·L^−1^ Brij^®^35, 0.7 mmol·L^−1^ linoleic acid, and increasing concentrations of carotenoids and/or α-tocopherol, rutin or chlorogenic acid (plus the control experiment without antioxidants) was transferred to a macro quartz spectrophotometer cell. At time zero, oxidation was initiated by addition of 40 μL iron solution to the reaction medium. Inhibition of linoleic acid oxidation by the carotenoids was evaluated at pH 4 with initiation by Fe^II^ (20 μmol·L^−1^) or metmyoglobin (0.5 μmol·L^−1^). At pH 5.8, inhibition of linoleic acid oxidation was evaluated only in the metmyoglobin model.

Lipid peroxidation was followed by measuring the concentration of conjugated dienes (CD: mainly hydroperoxides + minor amounts of alcohols) for about 90 min. Their absorbance was directly recorded by UV-VIS spectroscopy at 234 nm (baseline recorded on acetate buffer or phosphate buffer) [12,26]. The residual carotenoid concentration was simultaneously measured at the λ_max_ value in the visible range.

Kinetic curves of CD accumulation were plotted without antioxidant (control) and with increasing concentrations of antioxidant. Inhibition of linoleic acid peroxidation was measured by the delay in hydroperoxide accumulation: the *T/T*_0_ ratio was calculated for each antioxidant concentration, with *T*_0_ = time required to produce a fixed CD concentration (corresponding to a 0.5 increase in A (234 nm) after iron addition) in the control experiment (no antioxidant), and T = time required to produce the same CD concentration in the presence of the antioxidant. The *T/T*_0_ *vs.* initial antioxidant concentration was plotted for each antioxidant in the three models: MbFe^III^ at pH 4 and 5.8 and Fe^II^ at pH 4. The IC50 value was defined as the antioxidant concentration giving *T/T*_0_ = 0.5.

*Reduction of Ferrylmyoglobin by the Carotenoids.* The experimental procedure was adapted from previous studies [26]. Ferrylmyoglobin (MbFe^IV^=O) was first formed in a spectrophotometer cell (2 mL) by adding small volumes (60 μL) of a concentrated aqueous solution of H_2_O_2_ (2 mmol·L^−1^, final concentration in the cell = 60 μmol·L^−1^) to a 60 μmol·L^−1^ MbFe^III^ solution in a pH 7 phosphate buffer containing 4 mmol·L^−1^ Brij^®^35. Spectral changes featuring the conversion of MbFe^III^ (specific peak at 505 nm) into MbFe^IV^=O (specific peak at 590 nm) were recorded in the visible range until stability was achieved (2–3 min). Then, small volumes (about 50 μL) of a concentrated solution of carotenoid in MeOH/THF (1/1, v/v) were added (final concentrations of the carotenoids in the cell = 0 or 25 or 100 μmol·L^−1^) and the reduction of MbFe^IV^=O back to MbFe^III^ was monitored at 590 nm.

*Statistical analyses.* Each treatment was run in triplicate. The experimental results were expressed as means ± standard deviation. Differences between means were assessed either with the student *t*-test (when two means were compared) or using ANOVA followed by the *post hoc* analysis using Tukey-Kramer. A *P* ≤ 0.05 was considered statistically significant. All statistical analyses were performed using Statview software.

## Supplementary Files

Supplementary File 1
