# Supplementary material for: Interactions between Carotenoids from Marine Bacteria and Other Micronutrients: Impact on Stability and Antioxidant Activity
Source: Mar Drugs. 2015 Nov 19;13(11):7020–39. doi: 10.3390/md13117020 (PMC4663564; doi:10.3390/md13117020)
Supplement: Supplementary File 1 [file marinedrugs-13-07020-s001.docx]

**Supplementary Materials**


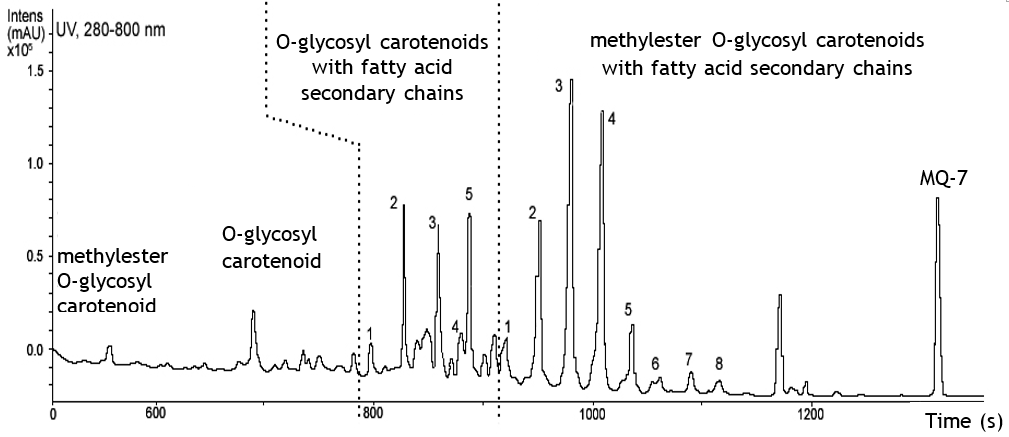


**Figure S1.** A UV-VIS chromatogram (200–800 nm) of the crude bacterial extract of HU36 containing carotenoids and MQ-7.

**Figure S2.** Iron-induced autoxidation of carotenoids HU36 (10 μmol·L^−1^) with and without MQ-7. The *Y*-axis displays the residual carotenoid concentration after 4 h of reaction at
pH 4. Initial iron concentrations were selected so as to maximize autoxidation after 4 h:
50 μmol·L^−1^ Fe^II^, 100 μmol·L^−1^ Fe^III^ and 0.5 μmol·L^−1^ MbFe^III^. Different letters indicate a significant difference between residual carotenoid concentrations (ANOVA followed by the Tukey-Kramer post-hoc test, *p* < 0.05).

|  |
| --- |
|  |
|  |

**Figure S3.** The percentage of residual β-carotene (**A**); lycopene (**B**) and lycopene + β-carotene (carotenes) (**C**) remaining during Fe^II^-induced autoxidation at pH 4. Fe^II^/carotenoid molar ratio = 0, 0.1, 0.5, 1.0 and 5.0.

|  |
| --- |
|  |
|  |

**Figure S4.** The percentage of residual β-carotene (**A**); lycopene (**B**) and lycopene + β-carotene (carotenes) (**C**) during Fe^III^-induced autoxidation at pH 4. Fe^III^/carotenoid molar ratio = 0, 0.1, 0.5, 1.0, 5.0 and 10.0.

| 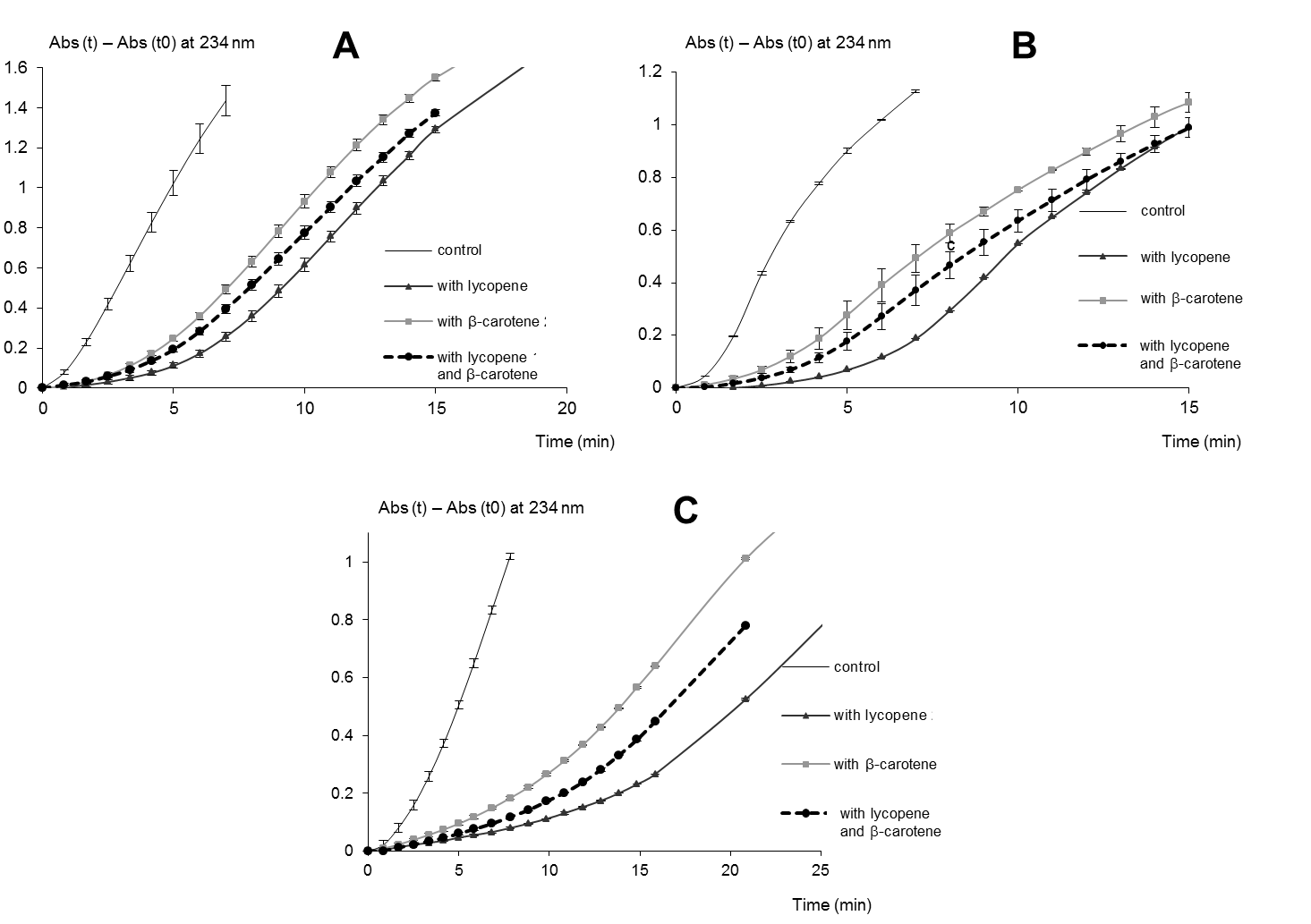 |
| --- |
| 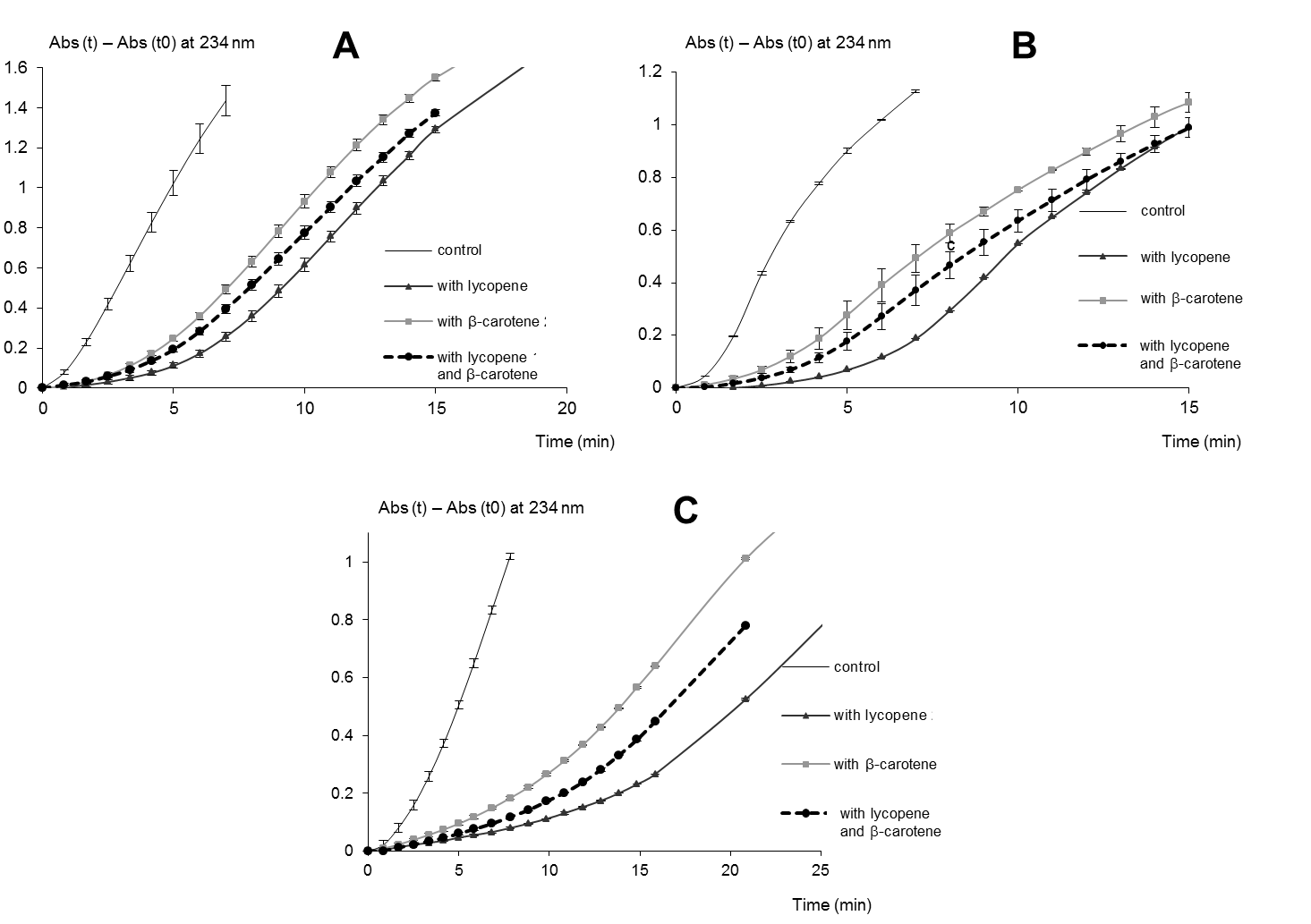 |
| 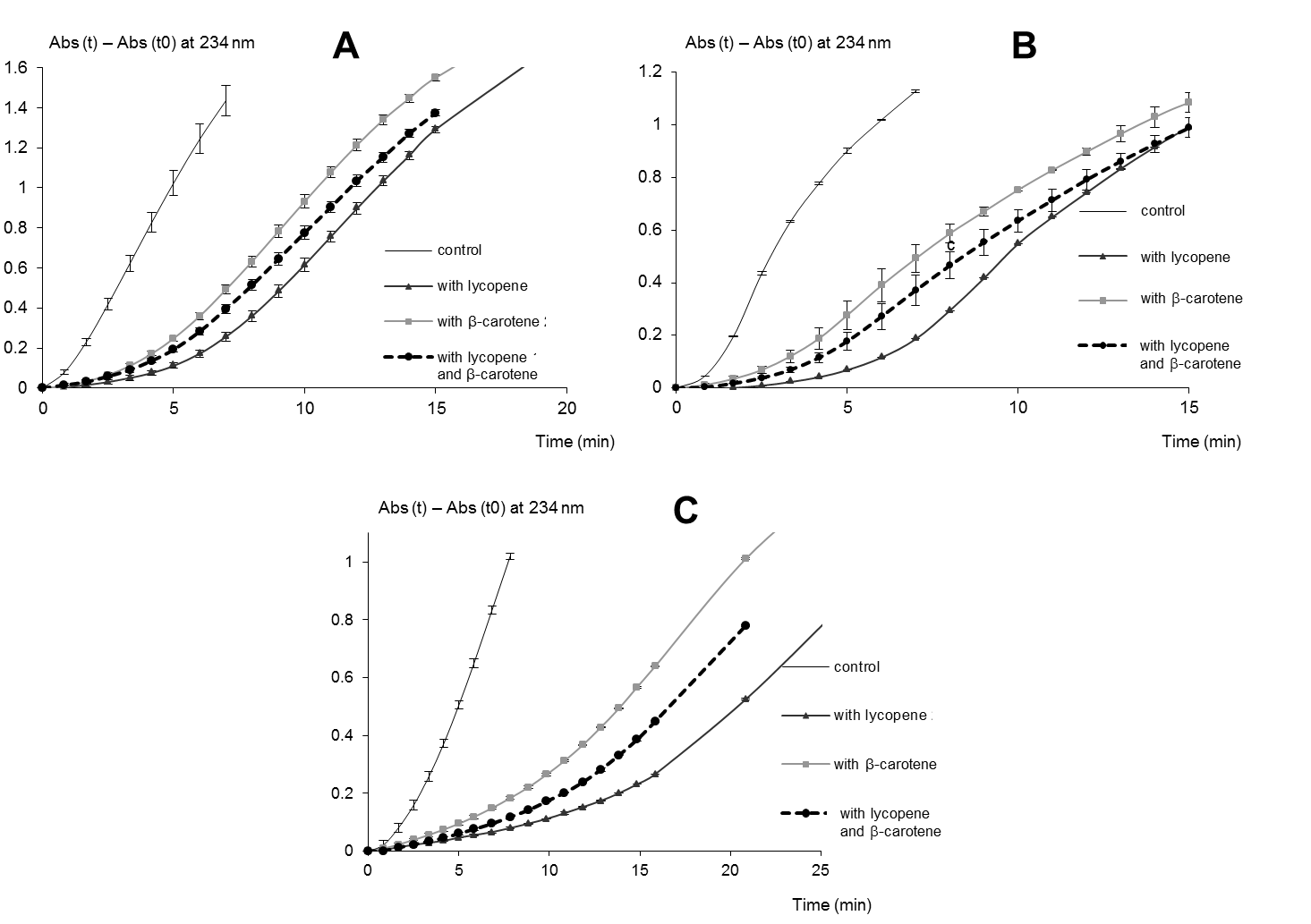 |

**Figure S5.** Inhibition of CD accumulation during linoleic acid peroxidation by lycopene
(1 μmol·L^−1^), β-carotene (1 μmol·L^−1^) and by a 1:1 mixture of both with the same total concentration of 2 μmol·L^−1^. Initiation by MbFe^III^, pH 5.8 (**A**) MbFe^III^, pH 4 (**B**) and Fe^II^, pH 4 (**C**).

**Figure S6.** Kinetic monitoring of ferrylmyoglobin formation (60 μmol·L^−1^ MbFe^III^ +
30 μmol·L^−1^ H_2_O_2_) at pH 7, and its subsequent reduction by antioxidants (100 μmol·L^−1^):
β-carotene (**a**); HU36 carotenoids (**b**); α-tocopherol (**c**); and chlorogenic acid (**d**).

© 2015 by the authors; licensee MDPI, Basel, Switzerland. This article is an open access article distributed under the terms and conditions of the Creative Commons Attribution license (http://creativecommons.org/licenses/by/4.0/).
